# Kikuchi-Fujimoto Disease: A Rare Presentation in a Young Male

**DOI:** 10.7759/cureus.55615

**Published:** 2024-03-06

**Authors:** Aishwarya K Kedar, Babaji Ghewade, Ulhas Jadhav, Pankaj Wagh, Vivek D Alone

**Affiliations:** 1 Pulmonary Medicine, Jawaharlal Nehru Medical College, Datta Meghe Institute of Higher Education & Research, Wardha, IND

**Keywords:** young adult, treatment, diagnosis, lymphadenopathy, histiocytic necrotizing lymphadenitis, kikuchi-fujimoto disease

## Abstract

Kikuchi-Fujimoto disease (KFD), also known as histiocytic necrotizing lymphadenitis, is a rare benign condition characterized by cervical lymphadenopathy and constitutional symptoms mimicking tuberculosis. We present the case of a 22-year-old male who presented with fever, dry cough, loss of appetite, multiple joint pains for 15 days, and loss of weight for one month. Physical examination revealed palpable cervical, occipital, axillary, and inguinal lymphadenopathy, and laboratory investigations were within normal limits except for raised erythrocyte sedimentation rate (ESR). Contrast-enhanced computed tomography (CECT) showed mediastinal lymphadenopathy with no pleuroparenchymal abnormality of the lung. Excision biopsy of a cervical lymph node confirmed necrotizing lymphadenitis consistent with KFD. The patient was treated with nonsteroidal anti-inflammatory drugs (NSAIDs) and glucocorticoids, resulting in the resolution of symptoms and regression of lymphadenopathy. This case signifies the importance of considering KFD in the differential diagnosis of lymphadenopathy and highlights the significance of histopathological evaluation for accurate diagnosis and management guidance.

## Introduction

Kikuchi-Fujimoto disease (KFD), also known as histiocytic necrotizing lymphadenitis, is a rare, self-limiting condition characterized by lymphadenopathy, fever, and constitutional symptoms. First described independently by Kikuchi and Fujimoto in Japan in 1972, this condition primarily affects young adults, with a slight predilection for females [1,2]. While its exact etiology remains elusive, it is believed to involve an aberrant immune response triggered by viral infections, particularly Epstein-Barr virus (EBV), human herpesvirus 6 (HHV-6), and cytomegalovirus (CMV) [3,4]. However, a definitive causal relationship has yet to be established.

Clinically, KFD often presents with nonspecific symptoms such as fever, night sweats, fatigue, and weight loss, mimicking infectious and autoimmune conditions, thereby posing a diagnostic challenge [5]. Additionally, patients may exhibit leukopenia, elevated inflammatory markers, and abnormal liver function tests [6]. The hallmark feature is a histopathological examination of lymph node biopsy specimens, revealing characteristic findings of necrotizing lymphadenitis with abundant karyorrhectic debris and histiocyte infiltration but the absence of neutrophils [7].

Treatment of KFD is largely supportive, with most cases resolving spontaneously within a few weeks to months. Symptomatic relief with nonsteroidal anti-inflammatory drugs (NSAIDs) and glucocorticoids may be warranted in severe or prolonged cases [8]. However, the overall prognosis is excellent, with recurrence being rare.

## Case presentation

A 22-year-old male presented to the outpatient department of the tertiary care hospital with complaints of fever, dry cough, and loss of appetite persisting for 15 days, along with multiple joint pains. During the medical history assessment, he disclosed a weight loss of 4 kilograms over one month. Notably, he denied any past occurrences of chest pain, breathlessness, hemoptysis, tuberculosis, or substance addiction. He had been self-medicating with antipyretics and analgesics for alleviating fever and joint discomfort.

Upon physical examination, the patient appeared debilitated but remained oriented to time, place, and person. Vital signs assessment revealed a temperature of 100.80°F, a pulse rate of 88 beats per minute, a respiration rate of 18 breaths per minute, a blood pressure reading of 110/80 mmHg, and an oxygen saturation level of 98%. On palpation, multiple cervical groups of lymph nodes, occipital lymph nodes, axillary lymph nodes, and inguinal lymph nodes were enlarged significantly. Hemogram and biochemical parameters from blood tests fell within normal ranges (Table 1) except for a raised ESR of 80 mm/hr.

**Table 1:** Laboratory investigation of the patient

Investigation name	Patient value	Reference value
Hemoglobin (Hb)	12.2	11-15 mg/dL
Packed cell volume (PCV)	24.9	32-46%
Red blood cell (RBC)	3.79	3.8-5.8 million/cumm
Mean corpuscular volume (MCV)	89	76-96 fl
Mean cell hemoglobin (MCH)	29.0	27-32 pg
Mean corpuscular hemoglobin concentration (MCHC)	32.5	31-35 gm/dl
Platelet count	2.60	1.5-4 lacs/cumm
Red cell distribution width (RDW)	18.2	1.-15%
Total leukocyte count (TLC)	5700	4000-11000/cumm
Differential leukocyte count		
Neutrophils	64	40-75%
Lymphocytes	30	20-45%
Eosinophils	03	1-6%
Monocytes	03	2-10%
Basophils	00	0-1%

**Figure 1:**
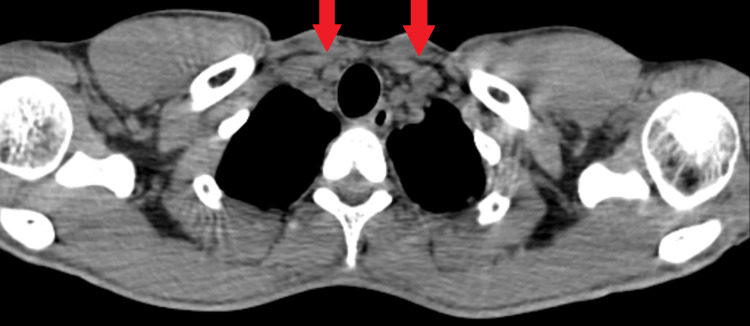
Shows mediastinal lymphadenopathy with no pleuroparenchymal abnormality

**Figure 2:**
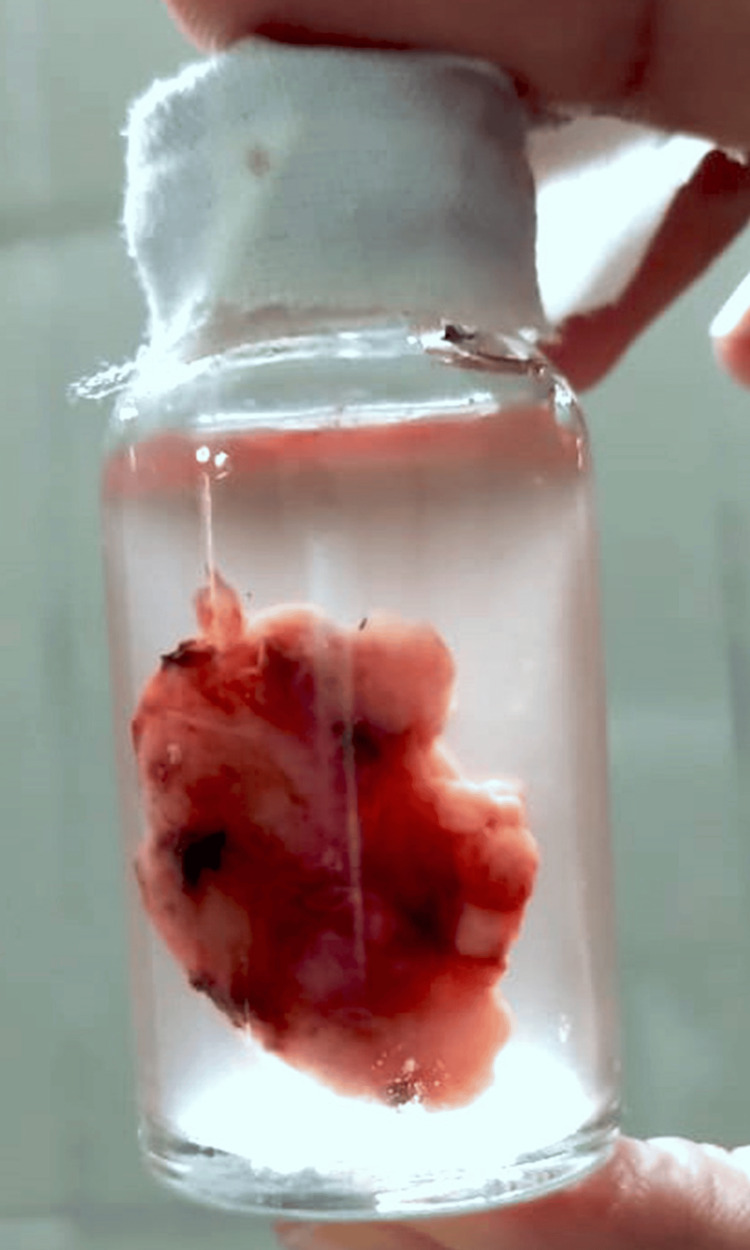
Shows left cervical lymph node which was sent for histopathological examination

**Figure 3:**
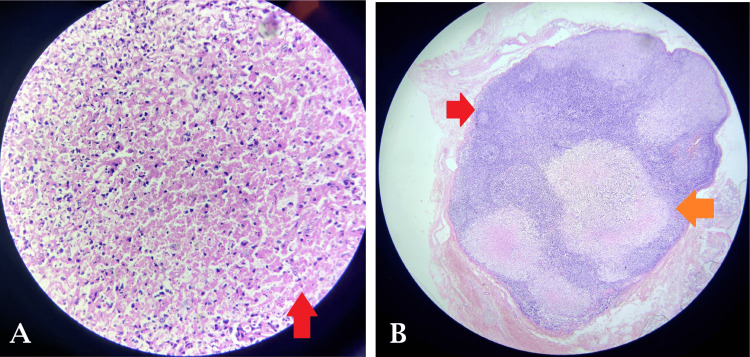
A) Sections studied shows abundant karyorrhectic debris, collections of mononuclear cells along with necrosis (H&E*High Power). B) Sections studied from lymph node biopsy shows focal, well-circumscribed, paracortical necrotizing lesions along with polymorphous population of lymphoid cells (H&E*low power)

The thorax's contrast-enhanced computed tomography (CECT) revealed mediastinal lymphadenopathy with no pleuroparenchymal abnormality (Figure 1). An excision biopsy of the left cervical lymph node was performed and sent for histopathological examination, which revealed necrotizing lymphadenitis with characteristic features indicative of Kikuchi-Fujimoto lymphadenitis (Figures 2-3). Consequently, a diagnosis of Kikuchi-Fujimoto lymphadenitis was established. The patient was started on treatment with NSAIDs and glucocorticoids, normalizing body temperature and reducing lymph node size.

## Discussion

KFD is a rare, benign condition characterized by histiocytic necrotizing lymphadenitis, predominantly affecting young individuals. While its exact etiology remains uncertain, various factors have been implicated, including viral infections, autoimmune mechanisms, and genetic predisposition [9,10]. Our case report adds to the existing literature by presenting a typical case of KFD in a young male, emphasizing the importance of clinical suspicion and histopathological confirmation for accurate diagnosis. The clinical presentation of KFD is often nonspecific, with fever, lymphadenopathy, and constitutional symptoms being common features. Differential diagnosis includes infectious etiologies such as viral lymphadenitis, tuberculosis, and autoimmune conditions like systemic lupus erythematosus (SLE) and lymphoma [11]. In our case, the patient presented with fever, dry cough, loss of appetite, weight loss, and multiple joint pains, which initially raised suspicion for infectious and rheumatological disorders. However, histopathological examination of the excised cervical lymph node revealed characteristic features consistent with KFD, confirming the diagnosis.

Laboratory investigations in KFD typically show nonspecific findings, with leukopenia, elevated inflammatory markers, and abnormal liver function tests being observed in some cases [6]. In our patient, hemogram and biochemical parameters were within normal limits except for a raised ESR of 80 mm/hr, highlighting the importance of histopathology in confirming the diagnosis. Imaging modalities such as CECT may demonstrate lymphadenopathy, aiding in the differential diagnosis, as seen in our case where the CECT thorax revealed mediastinal lymphadenopathy. Management of KFD is primarily supportive, with most cases resolving spontaneously within a few weeks to months. Symptomatic treatment with NSAIDs and glucocorticoids may be considered in severe or prolonged cases, although the evidence supporting their use remains limited [3]. In our case, initiation of NSAIDs and glucocorticoids resulted in the resolution of symptoms and regression of lymphadenopathy, consistent with previous reports.

## Conclusions

In conclusion, the presented case explains the diagnostic complexity of KFD, a rare entity characterized by histiocytic necrotizing lymphadenitis. Our patient's clinical course exemplifies the challenge of distinguishing KFD from other infectious and autoimmune conditions in young individuals presenting with fever and lymphadenopathy. Through histopathological examination of lymph node biopsy specimens, KFD was definitively diagnosed, emphasizing the crucial role of tissue analysis in confirming this condition. Treatment primarily consists of supportive measures, with NSAIDs and glucocorticoids offering symptomatic relief. In our patient, initiating this therapy resulted in clinical improvement and regression of lymphadenopathy, corroborating existing evidence. Overall, this case explains the importance of considering KFD in the differential diagnosis of lymphadenopathy and highlights the significance of histopathological evaluation in achieving an accurate diagnosis and guiding appropriate management strategies. Increased awareness of this condition among clinicians is imperative for ensuring timely recognition and optimal outcomes for affected patients.
